# De novo developed microsatellite markers in gill parasites of the genus *Dactylogyrus* (Monogenea): Revealing the phylogeographic pattern of population structure in the generalist parasite *Dactylogyrus vistulae*

**DOI:** 10.1002/ece3.8230

**Published:** 2021-11-10

**Authors:** Michal Benovics, Lenka Gettová, Andrea Šimková

**Affiliations:** ^1^ Department of Botany and Zoology Faculty of Science Masaryk University Brno Czech Republic

**Keywords:** Cyprinoidei, historical dispersion, host‐specific parasites, polymorphic markers, population genetics

## Abstract

Approaches using microsatellite markers are considered the gold standard for modern population genetic studies. However, although they have found application in research into various platyhelminth taxa, they remained substantially underutilized in the study of monogeneans. In the present study, a newly developed set of 24 microsatellite markers was used to investigate the genetic diversity of the generalist monogenean species *Dactylogyrus vistulae*. The analyzed parasite specimens were collected from 13 cyprinoid species from 11 sites in the Apennine and Balkan peninsulas. A total of 159 specimens were genotyped at each of the loci and the number of alleles per locus ranged from 2 to 16, with a mean number of 6.958 alleles per locus. Exceptionally high genetic diversity was observed among *D*. *vistulae* individuals in the southern Balkans (mean *N*_A_ per locus = 3.917), suggesting that generalist *D*. *vistulae* expanded from the south to the north in the Balkans and later into central Europe. The initial clustering analysis divided all investigated specimens into three major clusters; however, the results of the subsequent analyses revealed the existence of various subpopulations, suggesting that the population structure of *D*. *vistulae* is associated with the diversification of their cyprinoid hosts. In addition, the partition of the parasite population was observed in regions of the sympatric occurrence of two host species, indicating that these hosts may represent a barrier for gene flow, even for generalist parasite species.

## Graphical abstract

The application of a newly developed set of 24 microsatellite markers revealed a strong genetic structure among generalist monogenean parasites. The initial clustering analysis divided all investigated specimens into three major clusters; however, the results of the subsequent analyses revealed the existence of various subpopulations, suggesting that the population structure of *D*. vistulae is associated with the diversification of their cyprinoid hosts. In addition, a partition of the parasite population was observed in regions of the sympatric occurrence of two host species, indicating that these hosts may represent a barrier to gene flow, even for generalist parasite species.

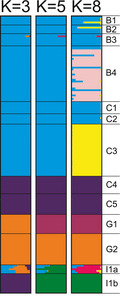

## 1 INTRODUCTION

The monogenean genus *Dactylogyrus* Diesing, 1850, is the most speciose taxon among Platyhelminthes. According to the checklist compiled by Gibson et al. (1996), this genus includes more than 900 nominal species, of which the majority are gill parasites of freshwater fish. However, this number is probably strongly underestimated considering the diversity of their common hosts—fish of the suborder Cyprinoidei (classification following the recent studies of Tan and Armbruster (2018), and Schönhuth et al. (2018)). The biogeographical distribution of *Dactylogyrus* concurs with the distribution of their cyprinoid hosts, as their occurrence in native species was documented in Africa, Asia, North America, and Europe (Hoffman, 1999; Pugachev et al., 2010; Řehulková et al., 2018). Each cyprinoid species potentially serves as a host for at least one *Dactylogyrus* species, and different lineages of *Dactylogyrus* usually parasitize divergent host lineages (Benovics et al., 2020; Benovics et al., 2018; Řehulková et al., 2020; Šimková et al., 2004, 2017). With respect to their hosts, *Dactylogyrus* parasites exhibit different levels of specificity. They range from strict specialists, which parasitize a single host species, through intermediate specialists parasitizing congeneric hosts and phylogenetic specialists parasitizing closely related hosts, to true generalists parasitizing phylogenetically distant host species, that is, hosts belonging to different families (Kuchta et al., 2020; Šimková et al., 2006).

*Dactylogyrus vistulae* Prost, 1967, is the most striking example of generalist *Dactylogyrus*, evidenced from 24 cyprinoid species of six genera in the Balkans and Central Europe (Benovics et al., 2018). This species was also reported from other European regions (e.g., Nordic countries; Koskivaara and Valtonen (1992), Southwest Europe; Seifertová et al. (2008), Apennine Peninsula; Benovics et al., 2021) and the Middle East (Aydogdu et al., 2001; Mhaisen & Abdul‐Ameer, 2019), suggesting that its distribution range might cover almost whole western palearctic ecoregion. In comparison with the majority of *Dactylogyrus* species, which generally parasitize only single host species or hosts from phylogenetically related lineages, the host range of *D*. *vistulae* encompasses a variety of phylogenetically divergent cyprinoid genera, that is, genera belonging to the families Cyprinidae and Leuciscidae (Benovics et al., 2018, 2021; Moravec, 2001), between which *D*. *vistulae* presumably often host‐switch (suggested by Benovics, Desdevises, Šanda, Vukić, & Šimková (2020). Benovics et al. (2018) investigated the molecular diversity of *D*. *vistulae* in the Balkans using partial 18S, ITS1, and partial 28S rDNA. They found 13 genetic variants in the Balkans and two genetic variants in the Czech Republic (representing Central European samples) representing different populations, that is, parasite populations associated with different host species or parasite populations associated with geographically distant populations of the same host species. Moreover, Benovics et al. (2018) showed a strong correlation between the genetic distances and geographical distances of *D*. *vistulae* populations, suggesting that geographical separation played a more critical role in the divergence of *D*. *vistulae* populations than their hosts’ phylogenetic relationships. Besides its remarkably wide host range and distribution range, *D*. *vistulae* is also easily distinguishable from other congeners due to its relatively large body size, and the size and simplicity of taxonomically important features (Pugachev et al., 2010), which promote this species as the optimal candidate for population genetic studies in dactylogyrid monogeneans.

Over the last few decades, approaches using microsatellite markers (together with mitochondrial DNA) have become the gold standard for most population genetic studies. These highly polymorphic short tandem repeats are, due to their unique characteristics (e.g., high allelic variance, codominance, and Mendelian inheritance), usually applied to infer gene flow rate, hybridization, or mating pattern on the intra‐ and interpopulation levels. Studies investigating interpopulation variability in monogeneans are often conducted using either morphometrics (Khang et al., 2016; Kmentová et al., 2016; Mariniello et al., 2004; Rahmouni et al., 2020; Rodríguez‐González et al., 2015; Vignon & Sasal, 2010), mitochondrial markers (Antonio Baeza et al., 2019; Huyse et al., 2017; Pettersen et al., 2021), or the combination of both methods (Huyse & Volckaert, 2002; Kmentová, Koblmüller, Van Steenberge, Artois, et al., 2020; Kmentová, Koblmüller, Van Steenberge, Raeymaekers, et al., 2020). However, until now, no genetic markers useful for revealing population structure in monogeneans have been identified for studies on dactylogyrids. Studying the population structure of parasites in association with their hosts—in our study, *Dactylogyrus vistulae* associated with cyprinoids mainly in Europe—may either reveal the geographical isolation between different cyprinoid species (or populations of the same cyprinoid species) or, alternatively, indicate secondary contacts between the hosts. Microsatellite markers are still underutilized in this platyhelminth group, and, to date, only one study designing primers for polymorphic repeats in gyrodactylids has been conducted by Faria et al. (2011). Therefore, the aim of this study was (1) to design a set of microsatellite primers for the widely distributed generalist monogenean species *D*. *vistulae* using next‐generation sequencing, and (2) to test their functionality with respect to revealing interpopulation genetic variability in order to investigate the geographical structure of the populations of this species.

## 2 MATERIAL AND METHODS

### 2.1 Material

**FIGURE 1:**
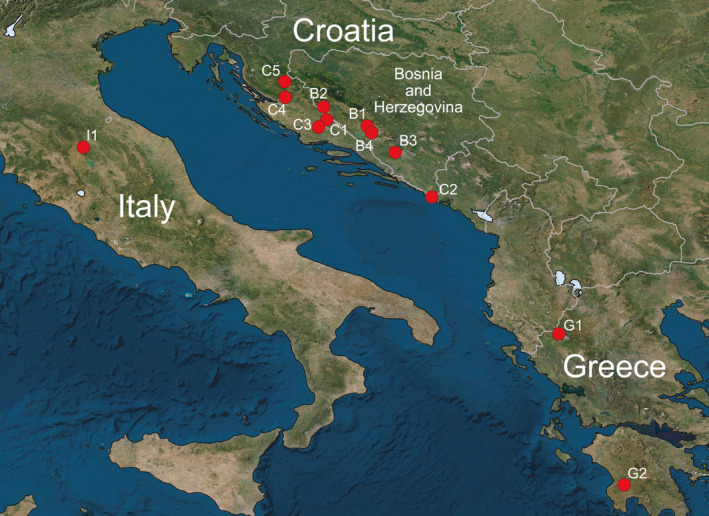
Map of collection sites in the Apennine Peninsula and the Balkans

To cover geographically distant regions within the distribution range of *D*. *vistulae*, parasites were collected from cyprinoid hosts at 29 sites across the Balkan and Apennine Peninsulas. The initial site selection followed the distribution of endemic cyprinoids, assuming that they will harbor endemic genetic lineages of parasites. As distribution range of many endemic species in the Balkans and Apennine Peninsulas is highly limited, and often covers only single river or river system (see Kottelat & Freyhof, 2007), the sampling sites are spread across the region. Moreover, we aimed to collect parasite individuals from sites where multiple endemic cyprinoids might occur. In the end, sites at which fewer than five *D*. *vistulae* individuals were collected were discarded from the final dataset; therefore, individuals only from 11 sites in the Balkans and one site in the Apennine Peninsula were included in this study (Figure 1). All included *D*. *vistulae* individuals were collected from 13 host species—six species of *Squalius*, four species of *Telestes*, two species of *Phoxinellus*, and *Chondrostoma phoxinus* (Table 1).

**TABLE 1:** List of host species investigated for presence of *Dactylogyrus vistulae* individuals including their respective collection sites with coordinates

ID	Host species	*N*	Country	Locality	Coordinates
B1	*Chondrostoma phoxinus*	5	Bosnia	Šujica, Šujičko Polje	43°49'41.43"N 17°10'48.20"E
B2	*Phoxinellus alepidotus*	5	Bosnia	Bosansko Grahovo, Korana river	44°10'37.00"N 16°23'03.61"E
B3	*Phoxinellus pseudalepidotus*	7	Bosnia	Lištica, Polog	43°20'32.09"N 17°41'37.04"E
B4	*Squalius tenellus*	32	Bosnia	Šujica	43°42'05.07"N 17°15'50.05"E
C1	*Squalius illyricus*	7	Croatia	Cetina river, Kosore	43°56'29.78"N 16°26'23.37"E
C2	*Squalius svallize*	6	Croatia	Konavočica, Grude	42°31'33.86"N 18°22'04.16"E
C3	*Telestes turskyi*	30	Croatia	rieka Čikola	43°48'22.09"N 16°17'24.53"E
C4	*Telestes croaticus*	10	Croatia	medzi Sveti Rok a Ličko Cerje, rieka Obsenica	44°21'03.64"N 15°40'40.00"E
C5	*Telestes fontinalis*	12	Croatia	Krbavsko polje, Laudonov gaj	44°38'14.33"N 15°40'05.65"E
G1	*Squalius prespensis*	11	Greece	Aoos, Kalithea	40°01'16.67"N 20°41'40.19"E
G2	*Squalius peloponensis*	18	Greece	Pamisos, Vasiliko	37°15'17.39"N 21°53'45.15"E
I1a	*Squalius squalus*	5	Italy	Torrente Cerfone, Intoppo	43°26'12.03"N 11°58'33.00"E
I1b	*Telestes muticellus*	11	Italy	Torrente Cerfone, Intoppo	43°26'12.03"N 11°58'33.00"E

Note

Codes labeled as ID are used in the following tables and figures. *N* = number of collected and analyzed *D*. *vistulae* individuals from the respective host.

### 2.2 Preparation of samples for NGS and identification of polymorphic tandem repeats

The whole genomic sequencing was performed from the pooled sample containing *D*. *vistulae* individuals collected from *Squalius cephalus* from the Dyje River (Czech Republic). Due to the focus of the paper on southern European regions with an intertwined geographic history, data on the *D*. *vistulae* population from the Czech Republic were omitted from further analyses. Genomic DNA was isolated using DNEasy Blood & Tissue Kit (Qiagen, Hilden, DE). Subsequently, library preparation using KAPA LTP Preparation Kit (Kapa Biosystems, Century City, SA) and sequencing on an Illumina Miseq platform (Illumina) using Miseq Reagent Kit v2 (Illumina) were provided by CEITEC (Masaryk University, Brno, CZ). The QDD (Meglécz et al., 2010) program was used to select 104 sequences with target microsatellites according to the following conditions: (i) The target microsatellites had at least seven repetitions, (ii) the length of the PCR product was between 100 and 300 bp, (iii) flanking regions did not contain a mononucleotide stretch of more than four bases, or any di‐hexa base pair motifs of more than 100 two repetitions, (iv) the annealing temperatures of primer pairs were optimized to fall between 57°C and 63°C, (v) microsatellites were not compound or interrupted, and (vi) selected sequences were not of fish origin based on BLAST results in the GenBank database.

### 2.3 PCR amplification, sequencing, and selection of microsatellite loci

Selected microsatellite primers were first tested separately in PCRs performed in a final volume of 10 µl, comprising 0.5 µl of template DNA, 0.2 mM dNTP, 1×PCR buffer, 2.0 mM MgCl_2_, 0.5 U Taq DNA polymerase (Invitrogen, Waltham, MA), and 0.2 µM of each of the forward and reverse primers. The conditions of the PCR were as follows: initial denaturation at 94°C (2 min), 35 cycles of denaturation at 94°C (60 s), annealing at 51–63°C (30 s), an extension at 72°C (2 min), and a final extension at 72°C (10 min). The PCR products were visualized by 2% agarose gel electrophoresis. Microsatellites that produced unreliable bands, or positive bands for the control sample represented by DNA of the fish host, were further excluded. The remaining microsatellites were tested under the same PCR conditions as described above, with the forward primer labeled by fluorescent dye (i.e., PET, NED, VIC, or FAM) and analyzed on an ABI PRISM 3130 Genetic Analyzer (Applied Biosystems, Foster City, CA) using 500 LIZ Size Standard (Applied Biosystems, Foster City, CA). Genotypes were scored using GeneMapper Software version 4.0 (Applied Biosystems, Foster City, CA). Finally, 24 polymorphic microsatellites were pooled into the five multiplex PCR sets. PCRs were carried out in a total volume of 10 µl using the Qiagen Multiplex PCR Kit (Qiagen, Hilden, DE) with 35 cycles and an annealing temperature of 52°C (Table 2).

**TABLE 2:** Description of de novo developed microsatellites markers for *Dactylogyrus vistulae* and conditions for multiplexing into five PCR sets

Locus	Primer sequence (5´−3´)	Repeat motif	Multiplex PCR sets		
			Set	μm	Dye
Dacty34	F: AGACATGCTCTGCTCACGAT	(AT)_9_	1	0.15	VIC
	R: TGATTCTAAGATGCGGGCAC				
Dacty36	F: CCGGTTCTTAGTTTAATGGGC	(AG)_9_	2	0.1	NED
	R: TGTGCACTGTTCCATCATGT				
Dacty38	F: CAAGTGGACTCGCAACAGAC	(AAT)_8_	1	0.15	PET
	R: CGGCACAATGAAAGGCTAT				
Dacty44	F: ACACGAGATGGTGCTTGATG	(AG)_10_	2	0.1	FAM
	R: TACGCTACAAGTGCTACAAGGA				
Dacty50	F: GCCCACGCTTGTCTTAACAT	(AG)_12_	1	0.15	VIC
	R: GTATGTCAACGCGCTCAATG				
Dacty52	F: AACACCATGAGAATGGAGCG	(AG)_8_	4	0.1	NED
	R: AATAGAGGGAGGGAAGGTGG				
Dacty54	F: CTTCCAAGGGACAACAGGAG	(AG)_7_	3	0.15	FAM
	R: TTGTCGATTTCAGCTCATGG				
Dacty64	F: AGACCAGCAAACGAAGTTGG	(AG)_7_	3	0.35	NED
	R: TTGGTCATTGCTAAGGTTTCC				
Dacty65	F: TTGCATTGCGTGATGGAC	(AC)_7_	4	0.25	PET
	R: TTGTACGTGTTGGTGCGATT				
Dacty66	F: TGCAGCATCGATTAAGTCTCA	(AG)_7_	2	0.15	VIC
	R: CCACTTGCATTCCCAGCTA				
Dacty67	F: TCATGAAAGAGAACGAAACGAA	(AT)_7_	2	0.4	PET
	R: TGGGTCAGACTGGATTTCCT				
Dacty68	F: AGGCATTTGCAACTCGATTA	(AGG)_7_	3	0.35	NED
	R: GCCAATCGCTGAGTTTGAA				
Dacty69	F: TAGAGGGAAGGCAAGTGTCC	(AAAC)_7_	3	0.15	VIC
	R: GCCATAGAAGCCAGCGAA				
Dacty70	F AATGCTGCCGAATTAACAGG	(ATC)_7_	3	0.25	PET
	R: TTGAGTGGGCTAGGTGTAGAAA				
Dacty73	F: AATTGAAGCGCTCCTCCG	(AG)_7_	4	0.15	FAM
	R: TCAATATCCAGTCTCGCAGC				
Dacty75	F CATGACCATGACAACCAACG	(CG)_6_	5	0.3	VIC
	R: ATGCACCACGCATCTATTTG				
Dacty79	F: GCAGTTTGTCCTGGCATTTC	(AT)_6_	4	0.2	FAM
	R: CACCAACTCGCCCTATGAGT				
Dacty84	F: AAGGTTGTAGCCTTGGTCAATC	(AG)_6_	5	0.2	FAM
	R: CAGCCAGTTGATCATCAGTTC				
Dacty85	F: GGTCGACGCTTCTCTTTGAT	(AG)_6_	1	0.3	PET
	R: GTCTCTAGAATTCGCCCGGA				
Dacty87	F: AAACATAGCCGCCAACCAG	(AGC)_6_	5	0.15	VIC
	R: TGTACACGAGCATTGAAGAGC				
Dacty92	F: CTTGCTTCAAACTCGGCTGT	(AAGGT)_6_	3	0.2	FAM
	R: CATGCATTCCCATCATTCAC				
Dacty93	F: ATTTGCCAATCTGTGCATGA	(ACTG)_6_	4	0.25	VIC
	R: GGGTTGGGTTGTTGGTAAAGT				
Dacty96	F: GGACAAGTTGAGTTGCTCGG	(AGC)_6_	5	0.25	PET
	R: GCGATACCATGTAGGGCAAG				
Dacty99	F: AACATGGAATAGGAGTGGGC	(AAC)_6_	1	0.15	FAM
	R: TCAATTGTACACGGACGAGC				

### 2.4 Genetic analyses

In total, 159 *D*. *vistulae* individuals were identified initially using morphological characters (shape and sizes of anchors, marginal hooks, connective bar, and sclerotized parts of male copulatory organ and vagina) and used in this study. Their species identities were evaluated by amplification of the partial genes coding 18S rDNA and 28S rDNA and entire ITS1 region (following Benovics et al. (2018)), and subsequent comparison to the sequences deposited in the GenBank. For microsatellite markers, the number of alleles (*N*_A_), the number of effective alleles (*N*_e_), Shannon's diversity index, the observed (*H*_O_) and expected (*H*_E_) heterozygosity, the fixation index (i.e., *F*, proportion of homozygotes), *F*_ST_ (i.e., genetic variance in allele frequencies), and the Nei‐distances (i.e., the number of eigen differences) were computed using the program GenAlEx v 6.5 (Peakall & Smouse, 2006, 2012). In order to test whether microsatellite markers are suitable for the identification of population structure in *D*. *vistulae* monogeneans, two methods were used: Bayesian clustering analysis, which was conducted in Structure v 2.3.4 (Falush et al., 2007; Pritchard et al., 2000) and multivariate Principal Coordinate Analysis (PCoA) implemented in GenAlEx v 6.5. An admixture model with expected uncorrelated allele frequencies was applied for the clustering analysis in Structure v 2.3.4. The expected number of clusters (*K*) was tested within the range of 1 to 15. Basically, *K* range was estimated on the basis that each parasite population was associated with an individual collection site (a total of 12 collection sites). However, to test whether further fragmentation occurs between sympatric hosts, or even within a single host population, the number of *K* was increased to 15. A series of ten independent runs with 10^7^ Markov chain Monte Carlo (MC^2^) iterations, the initial 10^6^ iterations discarded as a burn‐in, were conducted for each tested K. The most optimal K (i.e., with the highest likelihood) was selected using the Structure Harvester (Earl & vonHoldt, 2012; Evanno et al., 2005), and plot graphs were visualized using CLUMPAK (Kopelman et al., 2015).

## 3 RESULTS

### 3.1 Allelic diversity in microsatellite loci

From all candidate loci, which were selected according to the results of PCR amplification and sequencing tests, 24 were detected to be polymorphic and were chosen for further population analyses. The genetic diversity of each locus per collection site, including range of alleles, total number of alleles per locus (TN_A_), number of alleles per population (*N*_A_), and observed and expected allele heterozygosity per population (*H*_O_, *H*_E_), is presented in Table 3. The number of alleles per locus ranged from 2 (locus 75) to 16 (locus 73) with a mean number of 6.958 alleles per locus. With respect to specific geographic sites (representing different host species), the highest level of genetic polymorphism was recorded in *D*. *vistulae* individuals parasitizing northwestern Greek *Squalius prespensis* (site G1). Individuals from this site exhibited the highest mean heterozygosity across all loci (*H*_O_ = 0.453), the highest number of alleles per individual (*N*_A_ = 3.917), and the highest mean number of effective alleles (*N*_e_ = 2.662). The descriptive statistics indicating mean values for basic population parameters for all loci in respect to the collection sites are reported in Table 4. In general, each population exhibited low genetic variability and encompassed a high proportion of homozygotic individuals, that is, the majority of loci were monomorphic in almost all populations and only interpopulation allele lengths were observed. Homozygotic individuals were present at five of the 12 collection sites. The only locus that did not amplify across all individuals from a single collection site was locus 96 in G1. Nei‐distances and *F*_ST_ were computed for each population pair and are reported in Table 5. Nei‐distances ranged from 0.022 to 1.731, and the greatest distances were found between the G2 population and each of the C4 and C5 populations. Pairwise *F*_ST_ ranged from 0.103 to 0.959, and the greatest distances were observed between population C4 and the following populations: B1, B2, C1, C2, and C3. A similar pattern was revealed for the C5 population.

**TABLE 3:** Summary of basic population genetic statistical parameters for 24 microsatellite loci by collection site

Locus	Size (bp)	TNA	B1			B2			B3			B4			C1			C2			C3		
			*N*_A_	*H*_o_	*H*_e_	*N*_A_	*H*_o_	*H*_e_	*N*_A_	*H*_o_	*H*_e_	*N*_A_	*H*_o_	*H*_e_	*N*_A_	*H*_o_	*H*_e_	*N*_A_	*H*_o_	*H*_e_	*N*_A_	*H*_o_	*H*_e_
Dacty34	204–218	6	1	–	–	1	–	–	1	–	–	1	–	–	1	–	–	1	–	–	1	–	–
Dacty36	179–211	9	1	–	–	1	–	–	1	–	–	1	–	–	1	–	–	1	–	–	1	–	–
Dacty38	231–240	4	1	–	–	1	–	–	1	–	–	1	–	–	1	–	–	1	–	–	1	–	–
Dacty44	226–252	10	1	–	–	1	–	–	1	–	–	1	–	–	1	–	–	1	–	–	1	–	–
Dacty50	90–110	6	1	–	–	1	–	–	1	–	–	2	–	0.342	1	–	–	1	–	–	1	–	–
Dacty52	134–158	10	1	–	–	1	–	–	1	–	–	1	–	–	2	0.143	0.337	1	–	–	1	–	–
Dacty54	186–212	10	1	–	–	1	–	–	2	–	–	1	–	–	1	–	–	1	–	–	1	–	–
Dacty64	245–253	5	1	–	–	1	–	–	1	–	–	1	–	–	1	–	–	1	–	–	1	–	–
Dacty65	215–239	9	1	–	–	1	–	–	1	–	–	1	–	–	1	–	–	1	–	–	1	–	–
Dacty66	160–200	13	1	–	–	1	–	–	1	–	–	2	–	0.219	1	–	–	1	–	–	1	–	–
Dacty67	105–109	3	1	–	–	1	–	–	1	–	–	1	–	–	1	–	–	1	–	–	1	–	–
Dacty68	92–119	8	1	–	–	1	–	–	1	–	–	1	–	–	1	–	–	1	–	–	1	–	–
Dacty69	109–133	6	1	–	–	1	–	–	2	0.143	0.133	2	–	0.404	1	–	–	1	–	–	1	–	–
Dacty70	148–163	5	1	–	–	1	–	–	1	–	–	1	–	–	1	–	–	1	–	–	1	–	–
Dacty73	108–148	16	4	–	0.720	1	–	–	2	–	–	5	0.125	0.561	1	–	–	1	–	–	7	0.033	0.546
Dacty75	198–204	2	1	–	–	1	–	–	2	0.143	0.133	1	–	–	1	–	–	1	–	–	1	–	–
Dacty79	189–203	4	1	–	–	1	–	–	1	–	–	1	–	–	1	–	–	1	–	–	1	–	–
Dacty84	143–175	8	1	–	–	2	–	0.320	1	–	–	1	–	–	1	–	–	2	–	0.278	1	–	–
Dacty85	181–196	5	1	–	–	1	–	–	1	–	–	1	–	–	1	–	–	1	–	–	1	–	–
Dacty87	92–107	6	1	–	–	1	–	–	1	–	–	1	–	–	1	–	–	1	–	–	1	–	–
Dacty92	126–151	6	2	–	0.320	1	–	–	1	–	–	2	0.031	0.031	1	–	–	1	–	–	1	–	–
Dacty93	110–122	4	1	–	–	1	–	–	1	–	–	1	–	–	1	–	–	1	–	–	1	–	–
Dacty96	174–189	5	1	–	–	1	–	–	2	0.167	0.153	1	–	–	2	–	0.245	1	–	–	1	–	–
Dacty99	185–203	7	1	–	–	1	–	–	1	–	–	2	–	0.064	1	–	–	1	–	–	1	–	–

Locus	Size (bp)	TNA	C4			C5			G1			G2			I1			I1a			I1b		
			*N*_A_	*H*_o_	*H*_e_	*N*_A_	*H*_o_	*H*_e_	*N*_A_	*H*_o_	*H*_e_	*N*_A_	*H*_o_	*H*_e_	*N*_A_	*H*_o_	*H*_e_	*N*_A_	*H*_o_	*H*_e_	*N*_A_	*H*_o_	*H*_e_
Dacty34	204–218	6	1	–	–	1	–	–	4	0.667	0.667	1	–	–	3	–	0.461	2	–	0.320	2	–	0.463
Dacty36	179–211	9	2	–	0.180	1	–	–	2	0.091	0.087	1	–	–	6	0.125	0.504	5	0.400	0.760	1	–	–
Dacty38	231–240	4	1	–	–	1	–	–	3	0.545	0.483	1	–	–	2	0.063	0.061	2	0.200	0.180	1	–	–
Dacty44	226–252	10	1	–	–	1	–	–	7	0.909	0.814	1	–	–	4	0.200	0.504	3	0.600	0.540	1	–	–
Dacty50	90–110	6	1	–	–	1	–	–	1	–	–	2	0.056	0.054	2	–	0.430	1	–	–	1	–	–
Dacty52	134–158	10	1	–	–	1	–	–	8	0.636	0.843	1	–	–	4	–	0.484	3	–	0.560	1	–	–
Dacty54	186–212	10	1	–	–	1	–	–	6	0.909	0.793	1	–	–	4	–	0.484	3	–	0.560	1	–	–
Dacty64	245–253	5	1	–	–	1	–	–	4	0.455	0.550	1	–	–	2	0.063	0.170	2	0.200	0.420	1	–	–
Dacty65	215–239	9	1	–	–	1	–	–	7	0.727	0.756	1	–	–	1	–	–	1	–	–	1	–	–
Dacty66	160–200	13	1	–	–	1	–	–	9	0.727	0.826	3	0.056	0.106	4	0.125	0.482	3	0.400	0.540	1	–	–
Dacty67	105–109	3	1	–	–	2	–	0.153	3	0.125	0.570	2	0.111	0.346	2	0.125	0.219	2	0.400	0.480	1	–	–
Dacty68	92–119	8	1	–	–	2	–	0.375	7	0.909	0.789	1	–	–	3	0.125	0.420	3	0.400	0.340	1	–	–
Dacty69	109–133	6	1	–	–	1	–	–	4	0.364	0.318	3	0.444	0.647	2	–	0.430	1	–	–	1	–	–
Dacty70	148–163	5	1	–	–	1	–	–	4	0.364	0.384	1	–	–	2	0.063	0.061	2	0.200	0.180	1	–	–
Dacty73	108–148	16	2	–	0.420	3	–	0.569	4	0.273	0.566	1	–	–	5	0.063	0.494	4	0.200	0.660	1	–	–
Dacty75	198–204	2	1	–	–	1	–	–	1	–	–	1	–	‐	1	–	–	1	–	–	1	–	‐
Dacty79	189–203	4	1	–	–	1	–	–	3	0.455	0.574	1	–	–	2	0.063	0.061	1	–	–	2	0.091	0.087
Dacty84	143–175	8	1	–	–	1	–	–	3	0.545	0.417	1	–	–	6	0.125	0.699	5	0.400	0.600	2	‐	0.496
Dacty85	181–196	5	1	–	–	1	–	–	2	0.364	0.463	2	0.250	0.305	1	–	‐	1	–	–	1	–	–
Dacty87	92–107	6	1	‐	–	1	–	–	4	0.364	0.541	3	–	0.364	4	0.063	0.373	4	0.200	0.740	1	–	–
Dacty92	126–151	6	1	–	–	1	–	–	2	0.364	0.397	1	–	–	4	0.188	0.484	3	0.600	0.560	1	–	–
Dacty93	110–122	4	1	–	–	1	–	–	4	0.545	0.678	1	–	–	2	–	0.305	2	–	0.480	1	–	–
Dacty96	174–189	5	1	–	–	1	–	–	–	–	–	2	0.235	0.360	3	–	0.461	2	–	0.320	1	–	–
Dacty99	185–203	7	1	–	–	1	–	–	2	0.545	0.463	1	–	–	4	0.125	0.492	3	0.400	0.640	1	–	–

Note

Codes in the first row represent collection site IDs; TN_A_ = total number of alleles of locus; *N*_A_ = number of alleles at particular site; *H*_O_ = observed heterozygosity; *H*_E_ = estimated heterozygosity; I1a = data only for *D*. *vistulae* from *S*. *squalus*; I1b = data only for *D*. *vistulae* from *T*. *muticellus*.

**TABLE 4:** Descriptive statistics indicating mean values for basic parameters for all loci by each collection site

Pop	*N*	*N*_A_	*N*_e_	*I*	*H*_o_	*H*_e_	*F*
B1	5	1.167	1.127	0.076	0.000	0.043	1.000
B2	5	1.042	1.020	0.021	0.000	0.013	1.000
B3	7	1.208	1.063	0.075	0.019	0.045	0.351
B4	32	1.375	1.119	0.115	0.007	0.068	0.794
C1	7	1.083	1.035	0.039	0.006	0.024	0.788
C2	6	1.042	1.016	0.019	0.000	0.012	1.000
C3	30	1.250	1.050	0.049	0.001	0.023	0.939
C4	10	1.083	1.039	0.039	0.000	0.025	1.000
C5	12	1.167	1.088	0.075	0.000	0.046	1.000
G1	10	3.917	2.662	0.966	0.453	0.499	0.083
G2	18	1.417	1.171	0.153	0.048	0.091	0.424
I1a	5	2.458	1.911	0.636	0.192	0.370	0.443
I1b	11	1.125	1.081	0.064	0.004	0.044	0.651

Abbreviation: *F*, fixation index; *H*_E_, estimated heterozygosity; *H*_O_, observed heterozygosity; *I*, Shannon's diversity index; I1a, data only for *D*. *vistulae* from *S*. *squalus*; I1b, data only for *D*. *vistulae* from *T*. *muticellus*;*N*, total number of alleles; *N*_A_, number of alleles per individual; *N*_e_, effective number of alleles; Pop, collection site ID.

**TABLE 5:** Pairwise Nei‐distances and *F*_ST_ between individuals from each population pair

	B1	B2	B3	B4	C1	C2	C3	C4	C5	G1	G2	I1a	I1b
B1	–	0.231	0.226	0.263	0.195	0.194	0.415	0.939	0.890	0.467	0.819	0.354	0.815
B2	0.031	–	0.281	0.224	0.103	0.527	0.619	0.959	0.909	0.487	0.850	0.377	0.854
B3	0.068	0.084	–	0.197	0.281	0.278	0.364	0.850	0.809	0.477	0.765	0.356	0.768
B4	0.069	0.057	0.086	–	0.207	0.270	0.369	0.882	0.834	0.494	0.806	0.383	0.748
C1	0.033	0.005	0.085	0.059	–	0.322	0.442	0.946	0.897	0.482	0.843	0.363	0.834
C2	0.022	0.045	0.083	0.079	0.048	–	0.664	0.958	0.905	0.487	0.849	0.373	0.855
C3	0.055	0.060	0.117	0.120	0.080	0.070	–	0.957	0.909	0.484	0.841	0.388	0.838
C4	1.534	1.549	1.494	1.387	1.544	1.517	1.544	–	0.344	0.538	0.884	0.574	0.859
C5	1.363	1.384	1.346	1.236	1.379	1.330	1.380	0.072	–	0.523	0.866	0.561	0.800
G1	1.091	1.141	1.361	1.405	1.134	1.134	1.143	1.590	1.481	–	0.474	0.304	0.539
G2	1.233	1.280	1.312	1.279	1.286	1.255	1.273	1.731	1.716	1.284	–	0.506	0.828
I1a	0.391	0.415	0.482	0.509	0.399	0.400	0.442	1.149	1.152	1.014	1.181	–	0.447
I1b	0.873	0.888	0.880	0.776	0.883	0.889	0.882	0.951	0.860	1.703	1.345	0.794	–

Note

Dashes represent zero values; Nei‐distances are on the left side; *F*_ST_ are on the right side.

### 3.2 Population structure

**FIGURE 2:**
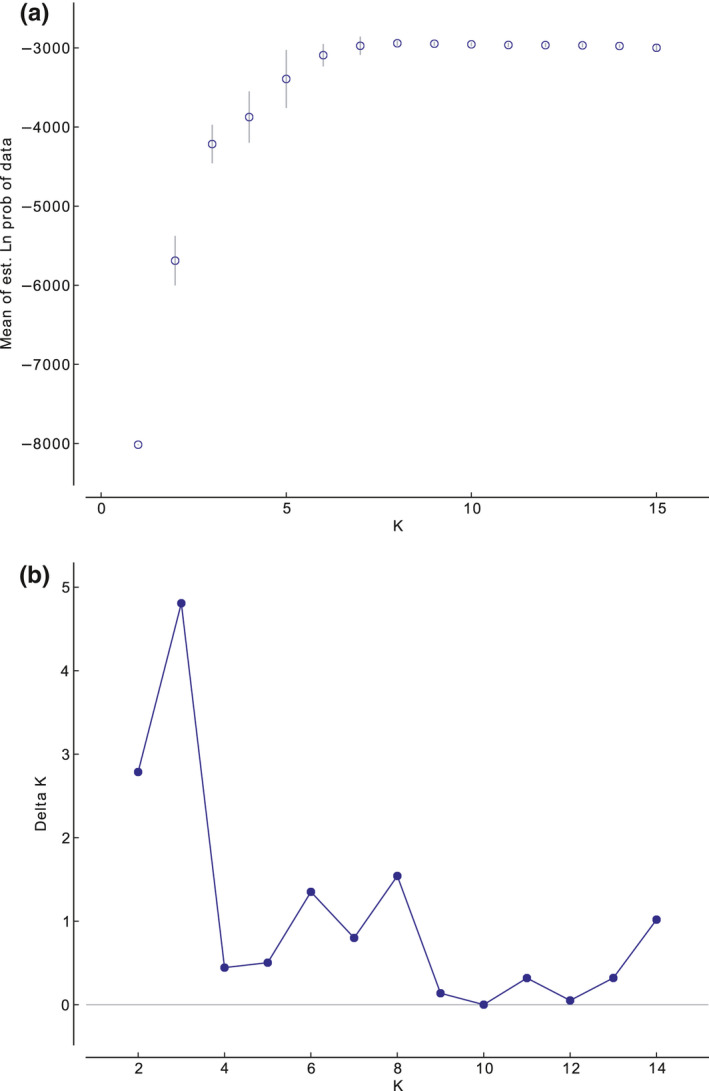
Mean estimated ln likelihood probabilities from 10 individual runs (a) and values of Δ*K* (b) for each tested number of clusters (*K* is shown on x‐axis) for the dataset analyzed in Structure v. 2.3.4. Δ*K* value indicates the most likely number of populations in the analyzed dataset

**FIGURE 3:**
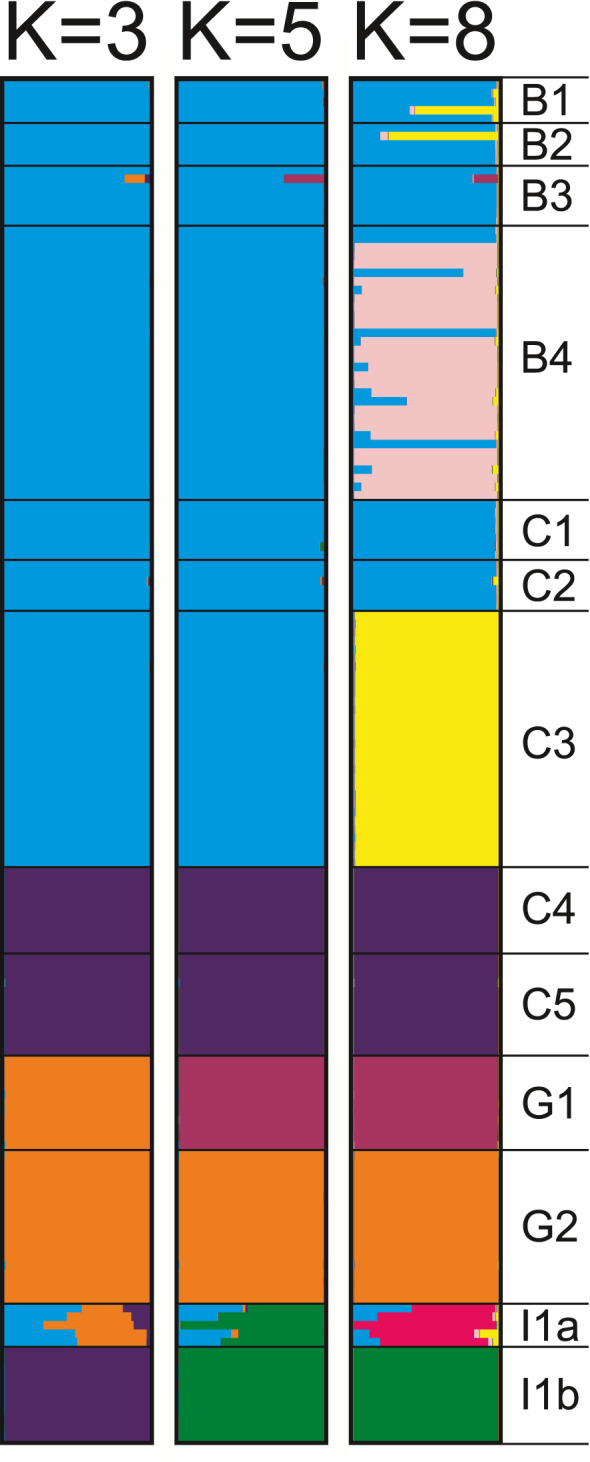
Results of Bayesian clustering analyses. Only bar plots for the estimated number of clusters (*K*) 3, 5, and 8 are shown. Each horizontal line within brackets represents one of 159 analyzed individuals. The estimated proportion of membership to a particular cluster is indicated by different colors. Labels on the right indicate collection sites of particular individuals

**FIGURE 4:**
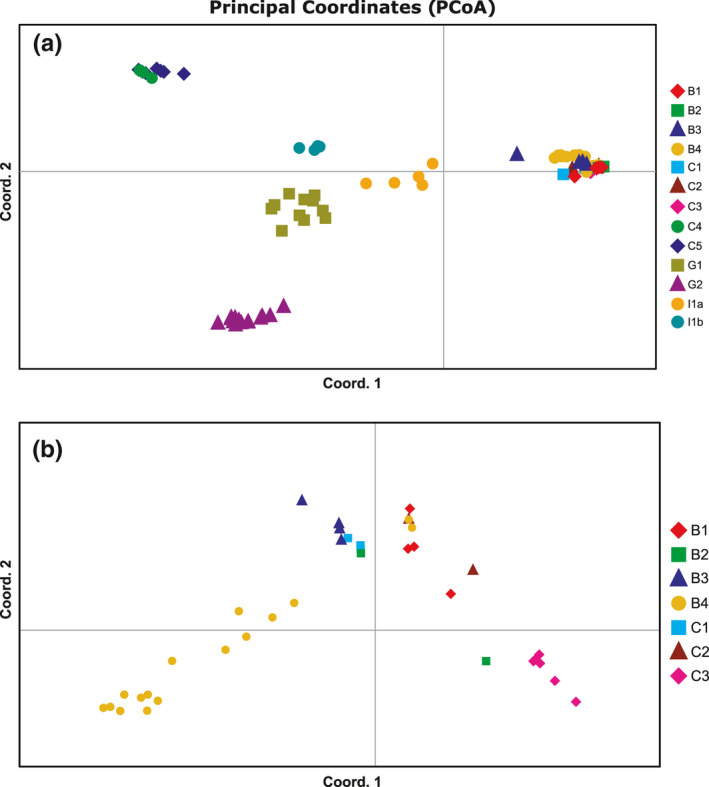
Results of PCoA computed in GenAlEx v. 6.5 for all analyzed populations (a), and for populations representing sites B1–B4, and C1–C3 only (b). Colored shapes indicate sites of collection of particular individuals. Percentage of variation explained by axes is presented in Table 6

The mean estimated likelihood of ten runs from all *K* tested by Bayesian clustering analyses peaked at *K* = 8, revealing a structured dataset encompassing eight population clusters (Figure 2a). However, the estimated Δ*K* peaked at *K* = 3 (Figure 2b), suggesting that major genetic structuration is observed for three estimated populations. Bar plots visualizing the proportion of the genome of each individual belonging to a particular cluster resulting from the Structure are shown for *K* = 3, *K* = 5, and *K* = 8 in Figure 3. The initial lower estimated K divided all investigated *D*. *vistulae* individuals into three major clusters (*K* = 3). The first one encompassed individuals collected from sites in Bosnia and Herzegovina (B1–B4), and southern Croatia (C1–C3). The second cluster encompassed all individuals from Greek sites G1 and G2. Finally, the third cluster encompassed individuals collected from sites in central Croatia (C4 and C5), and individuals parasitizing *T*. *muticellus* in Italy (termed as I1b). The *D*. *vistulae* individuals parasitizing *S*. *squalus* from the same site in Italy (I1a) were genetically ambiguously associated with all three clusters. Gradually increasing the number of estimated *K* first separated Greek *D*. *vistulae* individuals into two clusters, each associated with the respective collection site (G1 and G2), and later separated the Apennine population parasitizing *T*. *muticellus* (*K* = 5). In addition, the existence of three subpopulations was suggested among *D*. *vistulae* from sites B1–B4 and C1–C3; however, the results from both clustering analyses clearly document gene flow between these populations representing seven collection sites in Bosnia and Herzegovina, and Croatia (*K* = 8). At the higher estimated *K* (<7), clustering analysis divided the individuals from Italy into two separate subpopulations—each associated with the respective host species. The separation of I1 subpopulations also indicates genetic drift, whereas the observed heterozygosity in population I1b (*H*_O_ = 0.004) is significantly lower in comparison to population I1a (*H*_O_ = 0.192, Table 4). The results of PCoA were congruent with the structure revealed by Bayesian clustering analysis (i.e., the separation of populations C4, C5, G1, G2, and I1, and the grouping of individuals from sites B1–B4 together with C1–C3, Figure 4a). Moreover, the analysis also visibly separated the subpopulations from site I1 on the *x*‐axis. Subsequent PCoA including only individuals from the latter cluster in segments 2 and 3 supported the partial separation of subpopulations from B4 and C3, as was suggested by Bayesian clustering analysis with higher estimated K (Figure 4b).

**TABLE 6:** Percentage of variation explained by the first three axes using PCoA on microsatellite markers in Figure 4

Figure	Axis	1	2	3
A	%	38.57	19.76	9.22
	Cum %	38.57	58.33	67.55
B	%	40.32	13.81	9.53
	Cum %	40.32	54.13	63.66

## 4 DISCUSSION

The suitability of *D*. *vistulae* for population genetic studies of monogenean parasites was previously suggested by Benovics et al. (2018). Even though the microsatellite markers are commonly used in such studies, so far are underutilized in the assessing of genetic diversity of monogeneans. Therefore, in this paper, we present *de novo* developed microsatellite markers for dactylogyrids, which we applied in the interpopulation study of generalist *Dactylogyrus* species exhibiting a wide distributional range (including a wide range of host species).

We used 24 microsatellite markers to investigate the molecular diversity of populations of the generalist parasite *D*. *vistulae*. We found low allele variance in all studied markers at ten out of the 12 sites in the northeastern peri‐Mediterranean, where *D*. *vistulae* was collected from 13 host species. High allelic diversity was observed in individuals from the two remaining sites (two parasite populations); Aoos in northwest Greece, and the Cerfone Stream (Tiber drainage) in Italy.

**FIGURE 5:**
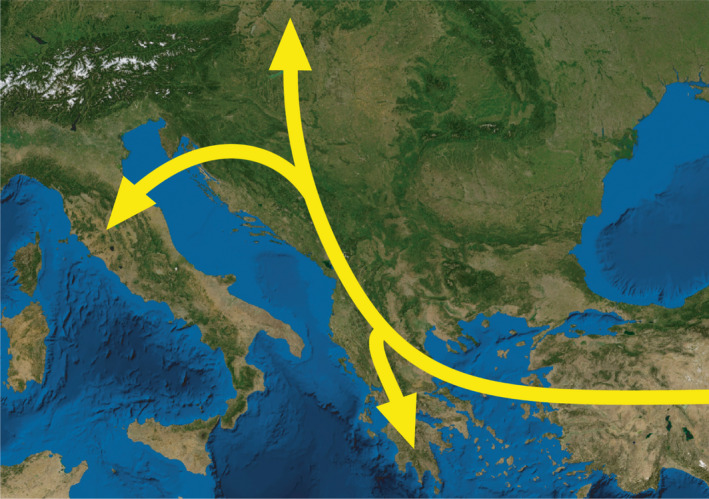
Hypothesized historical dispersion route proposed for *Dactylogyrus vistulae* in the northeastern peri‐Mediterranean based on observed genetic diversity

The surprisingly high observed genetic diversity in the Greek population might indicate either presence of exceptionally large population of *D*. *vistulae* in Aoos as a result of high abundance of suitable hosts in the region (expecting the same patterns of genetic variation as in the vertebrates (e.g., Hague & Routman, 2016) also in parasites), or a relatively more ancestral origin for this population than in the case of other Balkan populations. Assuming that the phylogeographic origin and historical dispersion of parasites are intimately linked with the phylogeography of their hosts, the latter is also supported by the historical dispersion route proposed for cyprinoids via the continental bridge dividing Paratethys and connecting the Balkan and Anatolian landmasses (Doadrio, 1990; Perea et al., 2010; Steininger & Rögl, 1984). In such a case, subsequent dispersion into the Balkan Peninsula occurred from south to north (Figure 5), and, therefore, the molecular diversity of *D*. *vistulae* is much lower in the north due to the founder's effect. A similar argument might be applied to explain the molecular diversity of *D*. *vistulae* in the Peloponnese. Even though the observed heterozygosity of Peloponnesian *D*. *vistulae* populations is higher than that of central Balkan populations, it might simply reflect the more ancestral diversification and subsequent isolation of parasite populations (or their host species) in the Peloponnese peninsula. The other factor that potentially impacted the formation of parasite populations in northwestern Greece is the historical formation of the Dessaretes lake system, which occurred in this region and played an essential role in the speciation of cyprinoids in the Balkans. This large connection of water bodies originated in the Pliocene and covered the area of all the present Great Lakes of the Balkan Peninsula, that is, Ohrid Lake (located on the border of Albania and North Macedonia), Prespa Lake (Albania, Greece, and North Macedonia), Mikri Prespa Lake (Albania and Greece), and Maliq Lake (Albania, evaporated during World War II) (Abell et al., 2008; Albrecht & Wilke, 2008; Bordon et al., 2009; Schultheiss et al., 2008; Sušnik et al., 2007; Wagner & Wilke, 2011). Later, after the closing of the Korca depression and connections between Paratethys and Dessaretes, the water level gradually decreased, promoting allopatric speciation in the freshwater fish fauna and leading to recent rich species diversity in the Great Lakes (Albrecht & Wilke, 2008; Steininger & Rögl, 1984). However, past underground hydrological connections between already divided lakes and neighboring drainages (as suggested by Albrecht and Wilke (2008)) facilitated the mixing of parasite populations between diversified host species and, therefore, gradually increased genetic diversity. At present, *S*. *prespensis* is an endemic species of lakes and rivers of the Prespa basin (Kottelat & Freyhof, 2007); therefore, we can assume that this species may share parasite communities genetically related to parasite communities of other endemic cyprinoids in the region of the former Dessaretes. However, thorough investigation of parasite communities, including, in particular, population analyses of widely distributed parasite species, is required to validate such a hypothesis.

In contrast to Greek populations, the high level of polymorphism in Italy can be explained by the syntopy of two co‐occurring hosts of *D*. *vistulae* in the region. In the Torrente Cerfone, *D*. *vistulae* individuals were collected from two cyprinoid species, *S*. *squalus* and *T*. *muticellus*. Clustering analyses revealed that each host species harbors a genetically divergent population of *D*. *vistulae* (Figure 3). However, the subsequent comparison of genetic variability between *D*. *vistulae* individuals parasitizing *S*. *squalus* and *D*. *vistulae* individuals parasitizing *T*. *muticellus* detected a high level of polymorphism only in the former population. A possible explanation for the observed difference in genetic polymorphism might be the putative origin of *D*. *vistulae* of the Apennine Peninsula. It has been hypothesized that during the Last Glacial Maximum, when the sea level drastically regressed (Bianco, 1990; Pielou, 1979; Waelbroeck et al., 2002), the expansion of the Po basin facilitated contact between freshwater fauna of the currently isolated Italian and Balkan river systems (Stefani et al., 2004; Waelbroeck et al., 2002). Such a connection would also explain the fact that cyprinoid fauna (e.g., *S*. *squalus* and its congeners) shows no or very little molecular divergence between species (or populations) living on both sides of the Adriatic Sea (Buj et al., 2010; Geiger et al., 2014; Perea et al., 2010). During this time, *S*. *squalus* (or its ancestor) dispersed together with other cyprinoids and their parasites from the Balkans to Apennine Peninsula (illustrated in the Figure 5). A high level of genetic polymorphism in *D*. *vistulae* may suggest that *S*. *squalus* served as a main host species for the dispersion of this parasite in the Apennine Peninsula, *where D*. *vistulae* later host‐switched to endemic species of *Telestes*. This hypothesis is also supported by the low genetic polymorphism in the *D*. *vistulae* population harbored by *T*. *muticellus* and by the calculated pairwise Nei‐distances and *F*_ST_. The population from *S*. *squalus* is genetically more similar to the central Balkan populations (Nei‐distances <0.509, *F*_ST_ < 0.388) than to the population from *T*. *muticellus* (Nei‐distance = 0.794, *F*_ST_ = 0.447, Table 4). Even though the historical dispersion of *D*. *vistulae* probably included host switching between species representing phylogenetically close genera (see also Benovics, Desdevises, Šanda, Vukić, & Šimková, 2020), the current hosts of *D*. *vistulae* in Torrente Cerfone apparently represent a barrier reducing the gene flow between parasite populations, even when host species live in syntopy, and biological requirements and habitats strongly overlap (see Kottelat and Freyhof (2007) for the biology and distribution of host species).

Initial clustering analyses separated all the studied populations into three major clusters, each essentially associated with the different ichthyogeographical regions (*sensu* Bianco, 1990). The clustering of individuals from the Apennine Peninsula and *D*. *vistulae* of the Balkan *T*. *croaticus* and *T*. *fontinalis* (sites C4 and C5, respectively) follows the range of the Padano‐Venetian district, which covers the region from the River Vomano in central Italy to the River Krka in Dalmatia. The range of this district essentially corresponds with the aforementioned Po basin during the glacial period. Moreover, these two *Telestes* species also represent a distant lineage from remaining congeners in the Balkans, whose ancestor diverged in the middle of the Miocene (Buj et al., 2017), which explains why the *D*. *vistulae* populations harbored by *T*. *croaticus* and *T*. *fontinalis* are divergent from other *D*. *vistulae* populations in the western Balkans. Thus, the second cluster encompassing individuals from four sites in Bosnia and Herzegovina and three southern Croatian sites is in accordance with the Dalmatian district's range, which corresponds with the distribution range of *S*. *svallize* (Bianco, 1990; Kottelat & Freyhof, 2007). The subsequent clustering analyses (Figure 3; K8, Figure 4b) separated individuals of *S*. *tenellus* (site B4) and *T*. *turskyi* (C3) into individual subpopulations; however, it also clearly indicated gene flow between *D*. *vistulae* populations sampled in Bosnia and Herzegovina, and those from Croatia. Both *S*. *tenellus* and *T*. *turskyi* are highly endemic with a strongly limited distribution range. While *S*. *tenellus* is primarily endemic to the Cetina River, some populations can also be found in nearby karstic streams; for example, *T*. *turskyi* is restricted to the Čikola River (Kottelat & Freyhof, 2007). In general, almost all endemic cyprinoid species in the Dalmatian district have an extremely limited distribution range, often limited to a single river stream in the karst (Bianco, 1990; Kottelat & Freyhof, 2007). Therefore, gene flow between populations of freshwater fish and between their parasites as well should be minimal. Nonetheless, the low genetic variance and the results of clustering analyses suggest the opposite. The partial mixing of parasite populations could result from recent contacts between hosts from geographically “isolated” regions via underground connections proposed by Palandačić et al. (2015). However, there is no doubt that the observed population structure is associated with the distribution of endemic hosts in the Balkans, and, from the low degree of heterozygosity we can assume that the subpopulations are of relatively recent origin.

Microsatellite markers represent a powerful molecular tool in population genetic studies of parasites. These polymorphic tandem repeats were previously employed in studies of the population structures of medically important human parasites (e.g., *Schistosoma haematobium*, Gower et al. (2011)). However, they are still strongly underutilized in studies of parasitic platyhelminthes in wildlife, as only a few papers focusing on cestodes (Bazsalovicsová et al., 2020; Luo et al., 2003; Štefka et al., 2009; Umhang et al., 2018) or digeneans (Criscione & Blouin, 2006; Criscione et al., 2006, 2011, 2020; Dar et al., 2015; Juhásová et al., 2016; Louhi et al., 2010; van Paridon et al., 2017; Valdivia et al., 2014; Vásquez et al., 2016) have so far been published. The above studies have shown that microsatellites, as molecular population markers, are more discriminative than rDNA and mtDNA. They have also demonstrated their usefulness in revealing the population genetic structure and historical migratory routes of parasites with a wide distribution range (Bazsalovicsová et al., 2020; Juhásová et al., 2016; Štefka et al., 2009), and migratory patterns of their hosts (Criscione et al., 2006). However, prior to our study, no population genetic studies utilizing microsatellites had been performed on monogeneans. Even though the primer set for the amplification of microsatellite loci was designed for *Gyrodactylus* (Faria et al., 2011), their only application was to assess modes of reproduction in this genus (Schelkle et al., 2012). The main reason for the scarcity of published microsatellite studies on monogeneans (and population markers in general) remains the collection of appropriate material for NGS. As the quantity and quality of the genomic DNA isolated from a small‐sized parasite may be insufficient, one option is to pool a considerable number of specimens (Vanhove et al., 2018). However, considering that monogeneans are generally small‐sized and that correct species identification is rather difficult without magnifying optical methods, there is a high risk of cross‐species contamination in the pooled samples, rendering the obtained genomic data challenging to process. The pooling technique was employed in this study because it used morphologically easily identifiable monogenean species. Therefore, we were able to provide and utilize the first set of *de novo* developed microsatellite markers that might open possibilities for population genetic studies in dactylogyrid monogeneans. Particularly in the highly diversified *Dactylogyrus*, which often exhibit extremely narrow host specificity, and thus their genetic population variability might reflect the remarkable variability of their hosts.

## CONFLICT OF INTEREST

The authors declare that they have no competing interests.

## AUTHOR CONTRIBUTIONS

**Michal Benovics:** Conceptualization (equal); Data curation (equal); Funding acquisition (supporting); Investigation (lead); Methodology (supporting); Project administration (supporting); Software (equal); Writing‐original draft (lead). **Lenka Gettová:** Conceptualization (equal); Data curation (equal); Investigation (supporting); Methodology (lead); Software (equal); Supervision (supporting); Validation (supporting); Writing‐review & editing (supporting). **Andrea Šimková:** Conceptualization (equal); Funding acquisition (lead); Project administration (lead); Supervision (lead); Writing‐review & editing (lead).

### OPEN RESEARCH BADGES

This article has earned an Open Data badge for making publicly available the digitally‐shareable data necessary to reproduce the reported results. The data is available at https://doi.org/10.5061/dryad.cnp5hqc5p.

## Data Availability

The microsatellite genotypes data are accessible in the Dryad data repository (https://doi.org/10.5061/dryad.51c59zw91).
